# Modelling Transdermal Permeation of Volatiles from Complex Product Formulations

**DOI:** 10.3390/pharmaceutics18020221

**Published:** 2026-02-09

**Authors:** Zhihao Zhong, Guoping Lian, Tao Chen, Yuan Yu

**Affiliations:** 1 Department of Chemical and Process Engineering, University of Surrey, Guildford GU2 7XH, UK; 2 College of Safety Science and Engineering, Nanjing Tech University, Nanjing 211816, China

**Keywords:** transdermal delivery, evaporation, permeation, stratum corneum, diffusion

## Abstract

**Background**: The evaporation of volatile ingredients from topical formulations strongly influences transdermal permeation and overall bioavailability, yet coupled evaporation–permeation dynamics are mostly simplified or neglected in existing models. **Methods**: We developed a mechanistic framework that couples Fickian gas-phase evaporation and transdermal permeation, both driven by the activity coefficients of volatiles. The model equations are implemented in a hybrid MATLAB–Python architecture with the volatile activity computed on-the-fly using UNIFAC and the gas-phase diffusivity calculated by the kinetic equation of Fuller–Schettler–Giddings (FSG). Initial validation used published IVPT data for 4-Tolunitrile and Nitrobenzene. **Results**: For 4-Tolunitrile, the FSG-based model estimated an initial evaporation coefficient of K_evap,i_ = 7.9348 × 10^−10^ mol·cm^−2^·s^−1^, and parameter optimization converged to 8.3929 × 10^−11^ mol·cm^−2^·s^−1^ (≈1/10 of the FSG estimate). The optimized model predicted an accumulation amount of 19.15% versus an experimental value of 16.97% in the receptor fluid (RF) at 24 h. For Nitrobenzene, the FSG initial estimation value of K_evap,i_ = 6.6480 × 10^−10^ mol·cm^−2^·s^−1^ was optimized to 8.1174 × 10^−11^ mol·cm^−2^·s^−1^ (≈1/8 of the FSG value), and the predicted amount of 24 h RF is 27.61% (experimental 23.19%). Both optimized K_evap,i_ values are roughly one order of magnitude lower than the initial FSG estimates, but >20× larger than Stokes–Einstein (SE)-derived values. Sensitivity scans show that further tuning of internal skin parameters (e.g., diffusion coefficient (D_SC,i_) and partition coefficient (P_SCw,i_)) produced only marginal improvements in RF prediction once K_evap,i_ was optimized. **Conclusions**: The coupled evaporation–permeation framework reproduces key IVPT kinetics for volatile solutes when the effective evaporation coefficient is calibrated. The kinetic-theory estimates (FSG-based) are a reasonable starting point, but typically overestimate the evaporation rate constant under finite-dose unoccluded IVPT conditions. By implementing the on-the-fly computation of volatile activity using UNIFAC, the approach is extensible to modelling transdermal permeation of volatiles from multicomponent/non-ideal formulations.

## 1. Introduction

Many topical products contain volatile compounds such as short-chain alcohols (e.g., ethanol and isopropanol), fragrance terpenes (e.g., limonene and geraniol), and small aromatic solvents. Their transdermal delivery presents a unique challenge in risk assessment and formulation design across dermatological, cosmetic, and environmental fields [1,2,3]. While the in vitro permeation test (IVPT) remains the regulatory standard for profiling dermal absorption prior to in vivo work [4,5,6], reproducibility becomes particularly difficult when dealing with volatile compounds. Unlike non-volatile permeants, volatile species undergo simultaneous evaporation and permeation, creating a dynamic competition that significantly complicates data interpretation and regulatory assessment [7,8]. This dynamic process is not only governed by the solute volatility, but also modulated by formulation [9]. Typical volatiles in topical products also span a wide physicochemical space, with vapour pressures ranging from well below 100 Pa to several kPa at ambient temperature, and LogP values from near-zero to >3, leading to significant contrasting effects on their activity and partitioning into the skin. For instance, short-chain alcohols tend to cause rapid early evaporation and transiently alter vehicle polarity [10]. Fragrance terpenes often have moderate vapour pressure with high lipophilicity, favouring skin partitioning over air-phase loss [11]. In particular, solvent evaporation can also affect bioavailability and cause transient supersaturation of the active ingredient, leading to drug precipitation. Furthermore, the direct mass loss of the volatile solute itself is a critical factor, and the present work focuses specifically on the latter.

IVPT can be performed under either ‘infinite dose’ or ‘finite dose’ conditions. For vehicles with volatile solutes and solvents, experimental emphasis has shifted towards studying finite doses under unoccluded conditions, to mimic the potential impact of evaporation on dermal absorption of products in use [12,13,14]. Mechanistic models, such as physiologically based pharmacokinetic (PBPK) models, are increasingly required to analyze the data and support more informed, faster, and cost-effective dermal absorption assessment [15,16,17].

Reported studies that incorporate evaporation into dermal absorption modelling remain limited, particularly regarding the impact on IVPT outcomes [18]. In unoccluded arrangements, volatile material that partitions to the gas phase may be incorrectly attributed entirely to evaporation or, conversely, to non-evaporative losses if volatilization is not measured. Under static (no forced-air) conditions, investigators frequently employ active capture or enrichment devices (e.g., filter traps; [19]) to quantify volatilization explicitly. By contrast, studies performed under dynamic (controlled-airflow) conditions have demonstrated substantially greater volatilization [20]. Notably, Kasting and colleagues varied airflow rates (≈10–100 mL min^−1^) and found that the fraction of analyte collected downstream increased with air flow, indicating markedly higher volatilization under forced-air conditions [21]. The mechanistic modelling pioneered by Kasting and co-authors parameterises volatilization using a mass-transfer coefficient (k), which relates to vapour pressure, molecular size, and environmental aerodynamic parameters (e.g., local airflow rate) [7,22,23,24]. The practical predictive value of such formulations is, however, often limited by incomplete characterization of experimental boundary conditions, including headspace volume, local ventilation/airflow, sample orientation and placement (bench versus fume hood), use of rotating platforms or static mounts. In practice, the absence of aerodynamic data commonly forces post hoc empirical calibration of the evaporation rate constants (or an equivalent airflow parameter). For example, fitting an airflow parameter was required to reproduce DEET evaporation and absorption data [21].

Despite the existence of the above models, some pivotal preceding work has attempted to provide a simpler modelling framework; for example, Deacon et al. proposed using the Stokes–Einstein (SE) equation to estimate evaporative diffusivity D_evap,i_ and simplifying the overall mass-transfer process as gas diffusion [25]. Subsequently, Zhang et al. developed an end-to-end workflow based on this work to simulate transdermal permeation under user-defined exposure conditions, providing a new solution that enables non-modelling specialists to perform transdermal permeation simulations [26].

However, this simplified model employed the ideal solution assumption (γ_l,i_ = 1) where activity equals unity, and it relies entirely on theoretical initial values without calibration or optimization to fit actual IVPT conditions. Consequently, a framework capable of handling the thermodynamic non-ideality at the vapour–liquid interface and supporting parameter optimization is required. It should also be noted that the SE relation is typically descriptive of diffusion in liquid media and not applicable to volatile diffusion in air. In addition, volatile evaporation also needs to overcome mass-transfer resistance at the air–liquid interface, which, if ignored, tends to overestimate the evaporation rate. This antagonistic interplay between the two effects complicates the accurate evaluation of evaporation from the mechanistic point of view.

In this study, the evaporation of volatile permeants is modelled by the mechanistic diffusion equation driven by the permeant’s vapour pressure at the liquid–air interface. This is then coupled with transdermal permeation by integrating with physiologically based pharmacokinetic (PBPK) modelling. In order to more accurately estimate the evaporation rate constant, the gas-phase diffusivity is calculated using the Fuller–Schettler–Giddings (FSG) method rather than the Stoke–Einstein relation [27,28]. The UNIFAC group-contribution method is used to dynamically calculate activity coefficients, which are essential for handling non-ideal solution thermodynamics and transient polarity shifts caused by the rapid solvent evaporation typical of multicomponent topical products.

By integrating activity-driven evaporation modelling with PBPK modelling, this approach provides a generic and scalable basis for future modelling of multicomponent formulations (e.g., co-solvents and emulsions). As an initial testing of the integrated evaporation PBPK model, we simulated the published IVPT data of volatile compounds 4-Tolunitrile and Nitrobenzene under finite dose and unoccluded conditions commissioned by the Cosmetics Europe task force [19,29]. We demonstrate that this approach can be applied for the effective extraction of evaporation parameters (e.g., K_evap,i_) and provides a generalized workflow for predicting the dermatokinetics of volatile ingredients, offering a robust alternative to airflow-dependent models.

## 2. Materials and Methods

Experimental data used for model validation were extracted from a previously published IVPT study [29]. For clarity, we summarize the experimental conditions that are relevant to the model comparison. Briefly, the IVPT was conducted with finite doses (10 µL·cm^−2^) of test chemicals applied under non-occluded conditions to human skin mounted in flow-through cells. The temperature was at 32 °C (305 K), and the relative humidity was not reported. The receptor fluid samples were collected over a 24 h period. After 24 h, the skin surface was washed, the stratum corneum was removed (tape-stripping), and the epidermis and dermis were separated. All compartments and receptor fluid were analyzed for radioactivity. Skin samples were dermatome-prepared to ~400 ± 50 µm thickness. For each chemical, the original studies used replicate discs from multiple donors (see original reports for full donor statistics). Full experimental protocols (chemical sourcing, purity, radio-labelling, and analytical methods) are reported in the cited publications.

The present model builds on previously published skin PBPK frameworks and incorporates modifications by recent modelling developments [30,31,32,33,34,35]. The skin is represented by a vertical sequence of tissue layers as principal transport routes, while including a localized pseudo-two-dimensional treatment for lateral exchange with hair follicles so that follicular (shunt) transport is captured without resorting to a full two-dimensional solver.

**Figure 1:**
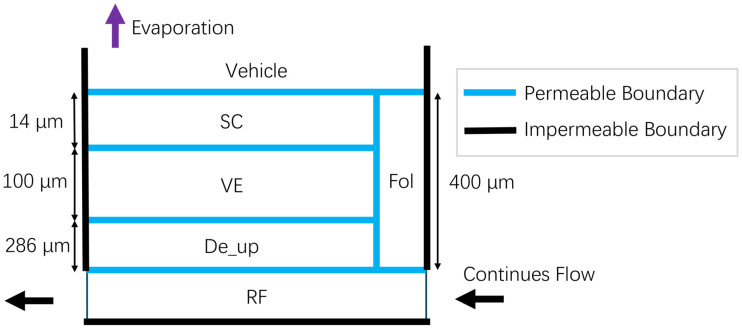
Diagram of the simulation model, which follows the setting of the reference paper.

The simulated skin tissue includes stratum corneum (SC), viable epidermis (VE), and dermis (De). For reproducibility, we report nominal example values used in this study (SC = 14 μm, VE = 100 μm, hair follicle (Fol) depth = 400 μm, and the De depth = 286 μm). Hair follicles were parameterized by depth and area fraction, then coupled to adjacent tissue layers, with lateral transport governed by the same diffusion-partition mechanisms used for vertical transport. The model schematic is shown in Figure 1. All compartments are treated as homogeneous continua for transport calculations. Key assumptions include: (i) homogeneous-layer representation (no explicit brick-and-mortar resolution), (ii) the hair follicular pathway modelled as an area-weighted conduit, and (iii) geometry that may be adjusted to match experimental sample thickness.

To facilitate interpretation, Table 1 summarizes the physicochemical properties (e.g., molecular weight, LogP, and vapour pressure) of the studied compounds that are relevant to both evaporation and transdermal permeation. These properties control gas-phase diffusivity and evaporative tendency as well as partitioning and diffusion within the skin; the calculation of these coefficients used in this model is detailed in Section S2 of the Supplementary Information, and all calculation results are also summarized in Table 1.

Consistent with the experimental protocol, the model was set to a finite dose regime. The initial application concentration and contact area (A) were set based on the cited data [29]. The receptor fluid (RF) was also set to a continuous-clearance mode to match the experimental setup.

The mass balance of a volatile solute in the vehicle (m_V,i_) thus evolves as competing evaporation (J_evap,i_) and skin-penetration (J_skin,i_) fluxes over the contact area A shown as Equation (1):

(1) $$\frac{d\left(C_{i}V\right)}{dt}=-J_{evap,i}A-J_{skin,i}A$$

(2) $$\frac{d\left(C_{i}V\right)}{dt}=V\frac{dC_{i}}{dt}+C_{i}\frac{dV}{dt}=-J_{evap,i}A-J_{skin,i}A$$

The volume of the vehicle follows the mass balance of the constituent permeant and solvent.

$$\frac{dV}{dt}=A\frac{dh}{dt}=\frac{A}{\rho }\sum \left(-J_{evap,i}-J_{skin,i}\right)M_{i}$$

With sufficiently low solute concentration in a non-volatile vehicle (PBS is temporarily treated as a non-volatile solvent), the vehicle volume (V) is approximated as constant. Equation (2) becomes.

(3) $$\frac{d\left(C_{i}V\right)}{dt}=V\frac{dC_{i}}{dt}=-J_{evap,i}A-J_{skin,i}A$$

where *m_V,i_* is solute mass in the vehicle (mol), *V* is vehicle volume (cm^3^), C_i_ is solute concentration (mol·cm^−3^), *A* is exposure area (cm^2^), *J_evap,i_* and J_skin,i_ are evaporation and transdermal permeation fluxes (mol·cm^−2^·s^−1^), ρ is the density of the vehicle, and *M_i_* is the molecular weight of *i*th solute.

The evaporation of volatiles, either solute or solvent, is modelled mechanistically as gas-phase transport governed by the surface vapor–liquid equilibrium activity. The flux (*J_evap,i_*) is defined by Fick’s law across the effective headspace height (h), as shown in Equation (4):

(4) $$J_{evap,i}=-D_{evap,i}\times \frac{m_{s,i}-m_{a,i}}{h}$$

The surface gas-phase concentration (m_s,i_) is derived from the liquid-phase state using the Modified Raoult’s Law combined with the Ideal Gas Law. This formulation linked the vapour pressure (P_v,i_) of the volatile to its activity (α_l,i_) and molar fraction (x_l,i_) in the vehicle

(5) $$m_{s,i}=\frac{P_{v,i}\cdot \alpha_{l,i}}{RT}$$

The activity (α_l,i_) is the thermodynamic driver for evaporation, defined by the product of the component’s molar fraction (x_l,i_) and its activity coefficient (γ_l,i_):

(6) $$\alpha_{l,i}=x_{l,i}\cdot \gamma_{l,i}$$

Crucially, γ_l,i_ accounts for non-ideal solution behaviour (where γ_l,i_ ≠ 1) and can be calculated dynamically using the UNIFAC method (see the section on thermodynamic implementation and coupling below).

By substituting Equation (6) into Equation (5) and assuming the ambient concentration m_a,i_ = 0, the gas-phase concentration M_s_ can be expressed in a form suitable for coupling with the UNIFAC implementation:

(7) $$m_{s,i}=\frac{P_{v,i}\cdot x_{l,i}\cdot \gamma_{l,i}}{RT}$$

Substituting Equation (7) into Equation (4) yields the mechanistic flux equation:

(8) $$J_{evap,i}=-D_{evap,i}\left(\frac{P_{v,i}\;x_{l,i}\gamma_{l,i}}{RT}\right)\frac{1}{h}$$

In previous studies [15,16], volatile diffusivity in air was calculated from the SE equation, which was known to apply to diffusion in liquid, not to diffusion in gas. Here, the D_evap,i_ in Equation (8) is proposed to be calculated by using the Fuller–Schettler–Giddings (FSG) equation based on the kinetic theory [27,28]:

(9) $$D_{evap,i}=\frac{{0.00143T}^{1.75}\sqrt{\frac{1}{{MW}_{i}}+\frac{1}{{MW}_{air}}}}{P{\left(V_{i}^{\frac{1}{3}}+V_{air}^{\frac{1}{3}}\right)}^{2}}$$

where D_evap,i_ is the binary diffusion coefficient of species *i* in air, in cm^2^·s^−1^. T is the absolute temperature, in K. P is the total pressure, in atm (normally P = 1 atm). MW_i_ and MW_air_ are the molar mass of species *i* and air (typically 28.97 in g·mol^−1^), respectively; V_i_ is the Fuller diffusion volume of species *i*, in cm^3^·mol^−1^, obtained by summing the diffusion volume contributions of all atoms or functional groups in the molecule (here, only the following groups are used: C:16.5, H:1.98, O:5.48, N:5.69 in cm^3^·mol^−1^). In addition, to account for the increased effective molecular volume associated with aromatic π-electron delocalization and ring rigidity, an empirical aromatic-ring correction is applied. Specifically, for each aromatic ring detected in the molecular structure, an additional contribution of 20.2 cm^3^·mol^−1^ is added to V_i_. And V_air_ is the diffusion volume of air (commonly 20.1), in cm^3^·mol^−1^.

For optimization, we define a lumped effective evaporation coefficient, K_evap,i_, as the product of the effective diffusivity from FSG theory and the vapour pressure (P_v,i_):

(10) $$K_{evap,i}=\frac{D_{evap,i}\cdot P_{v,i}}{hRT}$$

This simplifies the flux equation to the form implemented for calibration is shown in Equation (11):

(11) $$J_{evap,i}=-K_{evap,i}\alpha_{l,i}$$

where the physical units are: J_evap,i_ (mol·cm^−2^·s^−1^); K_evap,i_ (mol·cm^−2^·s^−1^); x_l,i_ and α_l,i_ (dimensionless); T (K); and h (cm). The simulation temperature is set to 305.15 K (32.0 °C), a value that was reported for the experiments to approximate the physiological warmth of the skin surface. For dimensional consistency (i.e., for P_v,i_/(R T) to yield units of mol·cm^−3^), the gas constant R must be used in units of Pa·cm^3^·mol^−1^·K^−1^ (approx. 8.314 × 10^6^).

For clarity, we note two practical choices: (i) the activity coefficient model used is UNIFAC, obtained from its implementation in the Python (version 3.13.5, distributed via Anaconda) thermo library (version 0.4.1), and (ii) all evaporation-related quantities reported are normalized per unit area (per cm^2^, as in the reference paper, the exposure area is 1 cm^2^). The diffusion path length *h* of volatiles in the air phase is set to 2 cm, which corresponds to the typical donor-chamber length (i.e., the distance from the formulation surface to the surrounding ambient) in standard IVPT setups. In this way, the model treats the air phase in a quasi-static manner (fixed *h*) and does not explicitly resolve local airflow, transient ventilation, or turbulence.

Calibration was performed using MATLAB (R2024b, Update 6, The MathWorks, Inc., Natick, MA, USA)’s lsqnonlin solver (trust-region reflective algorithm) to minimize a composite objective function within predefined parameter bounds. The objective function consists of the residual sum of squares (RSS) of the data points; the complete formulation is provided in Supplementary Methods (S3). After the single-parameter identification of K_evap,i_, a high-resolution local one-dimensional scan (NPts = 150, spanning ± 50% around the best-fit value) was conducted to confirm the presence of a well-defined MSE minimum. Subsequently, the preliminarily optimized K_evap,i_ was fixed, and single-parameter optimizations were performed for the SC properties (D_SC,i_ and P_SCw,i_) to assess whether further improvement in the fit could be achieved. All grid configurations and diagnostic data are provided in the Supplementary Information.

Numerical solution and acceptance criteria follow standard practice for stiff transport equation systems. The coupled ODE system is integrated with MATLAB’s ode15s using adaptive time stepping. The solver tolerances used for all simulations and sensitivity checks were RelTol = 10^−4^ and AbsTol = 10^−6^, with a non-negative constraint applied to all concentrations. Runs with solver failures or non-physical states are inspected and excluded.

Model inputs are based on the data provenance derived from the cited IVPT study. Receptor-fluid time series and the glass-tube pre-recovery dataset (open-headspace, 4 h) are from the same cited paper [19,29]. The selected compounds 4-tolunitrile and nitrobenzene have molecular weights of 117.15 and 123.11 Da, respectively, and LogP values of 2.09 and 1.85. In the preliminary open-headspace experiments, their 4 h mass losses were 81% and 89%, respectively. In the 24 h IVPT experiments, the corresponding mass losses were 80.40% and 66.87%.

All extended derivations, the full UNIFAC parameterization, detailed QSPR formula, prior-value calculations, including estimates for the initial calculated value of D_evap,i_, calibration settings, and complete scan/map outputs are collected in the Supplementary Sections S1–S3.

## 3. Thermodynamic Implementation and Coupling

To capture the non-ideal mixing behaviour of solute and solvent during the finite-dose evaporation process, the model integrates the UNIFAC activity coefficient method via a hybrid computational framework. Unlike traditional models that rely on static look-up tables or assume ideal solution behaviour (γ_l,i_ = 1), a dynamic MATLAB-Python interface has been developed to compute activity coefficients γ_l,i_ on-the-fly at each integration time-step. This ensures that the thermodynamic state remains consistent even as temperature varies or composition shifts due to solvent and solute depletion in the vehicle.

The implementation workflow is as follows: At each step of the MATLAB ODE solver (ode15s), the instantaneous molar concentrations are converted into a molar fraction vector (x). This vector, along with CAS registry numbers, is passed to the Thermo Scientific library (version 0.4.1) within a Python environment [36,37,38]. The Python script executes a hierarchical retrieval strategy: it first attempts to automatically deduce UNIFAC group assignments via the DDBST database. Crucially, to ensure generalizability across a wide range of formulations, we implemented a JSON-based ‘override mechanism’. This allows for the manual specification of functional groups for novel chemical entities (NCEs) or excipients not present in standard databases, making the model agnostic to the specific permeant.

Furthermore, to guarantee numerical robustness, the interface includes comprehensive error-handling mechanisms. These safeguards prevent solver failure during unphysical transient states (e.g., negative concentrations during iterative steps) by trapping convergence errors and maintaining calculation stability. The computed activity coefficients are returned to MATLAB via JSON to update the Modified Raoult’s Law, thereby driving the evaporation flux with high physical fidelity.

## 4. Results

Published IVPT data for 4-Tolunitrile and Nitrobenzene are used as case studies to evaluate the accuracy and robustness of the model in handling highly volatile compounds. The open-top experiments (glass tubes under ambient air exposure) reported mass losses of approximately 81% and 89% at 4 h for 4-tolunitrile and nitrobenzene, respectively, while the cited IVPT study reported cumulative mass losses of 80.40% and 66.87% at 24 h [19,29]. These results indicate that both compounds exhibit substantial volatility; therefore, we attribute the majority of the mass loss observed in the IVPT study to solute evaporation. This assumption is consistent with their physicochemical profiles (Table 1), in particular their relatively high vapour pressures and moderate lipophilicity (LogP).

The lumped mass-transfer coefficient, K_evap,i_ (units: mol·cm^−2^·s^−1^), is established as the primary calibration parameter for modelling evaporation, governed by Fick’s law of gas-phase transport. To ensure a mechanistic estimation of the gas-phase diffusion rate, the evaporation diffusivity D_evap,i_ is calculated using the Fuller–Schettler–Giddings (FSG) method (as shown in Equation (9)), rather than the Stokes–Einstein (SE) relation, which is typically limited to liquid-phase diffusion. This D_evap,i_ is subsequently substituted into the K_evap,i_ calculation (Equation (11)).

We first performed forward simulations using the FSG-based initial calculated value for K_evap,i_. The Fuller diffusion volume (V_i_) for each compound was determined by summing atomic/functional group contributions as described above. Temperature is set to T = 305.15 K, and the volatile diffusion path length is set to h = 2 cm to mimic the IVPT experimental configuration.

For 4-Tolunitrile (C_8_H_7_N, MW = 117.15 g·mol^−1^), the Fuller diffusion volume V_i_ is 171.7500 cm^3^·mol^−1^ and the vapour pressure P_v,i_ is 41.7298 Pa. Application of Equation (9) yielded the gas-phase diffusivity D_evap,i_ = 9.6481 × 10^−2^ cm^2^·s^−1^. Substituting this D_evap,i_ into Equation (11) (evaporation path length h = 2 cm) resulted in an initial calculated value of K_evap,i_ = 7.9348 × 10^−10^ mol·cm^−2^·s^−1^. For comparison, the SE equation at the same *T* and h gives D_SEevap,i_ = 4.0501 × 10−4 cm^2^·s^−1^ and K_SEevap,i_ = 3.3309 × 10^−12^ mol·cm^−2^·s^−1^, both more than two orders of magnitude lower than the FSG-based estimates.

Similarly, for Nitrobenzene (C_6_H_5_NO_2_), MW = 123.11 g·mol^−1^), the Fuller diffusion volume V_i_ was 145.7500 cm^3^·mol^−1^ and P_v,i_ = 32.6639 Pa. The FSG estimate produced D_evap,i_ = 1.0327 × 10^−1^ cm^2^·s^−1^ and an initial K_evap,i_ = 6.6480 × 10^−10^ mol·cm^−2^·s^−1^. The SE equation gives D_SEevap,i_ = 3.9837 × 10^−4^ cm^2^·s^−1^ and K_SEevap,i_ = 2.5645 × 10^−12^ mol·cm^−2^·s^−1^, again more than two orders of magnitude lower than the FSG values.

**Figure 2:**
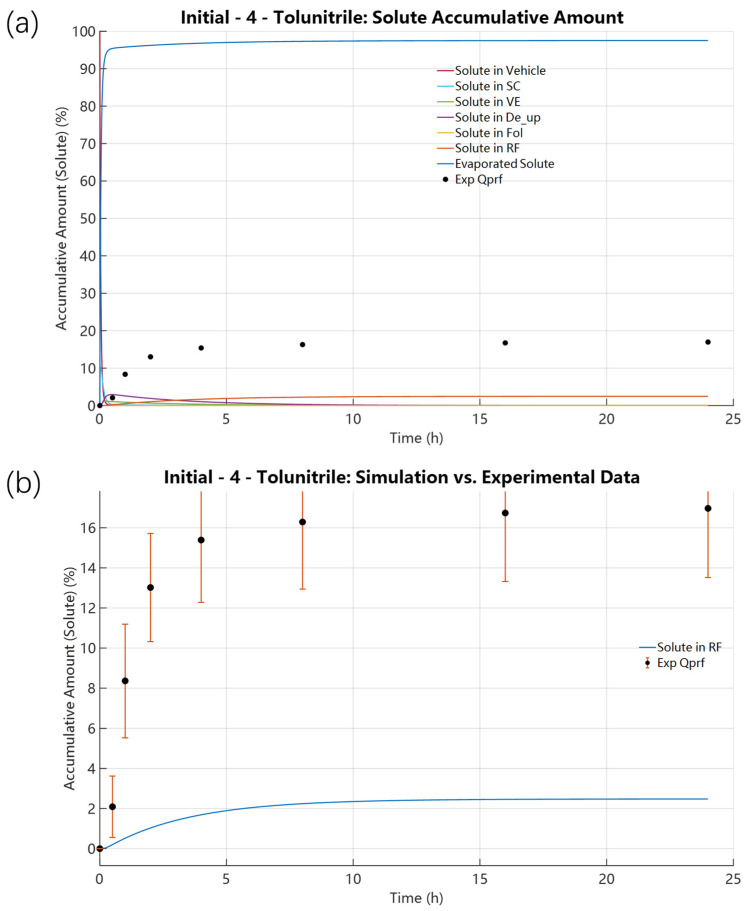
Solute distributions under initial calculated value of 4-Tolunitrile: K_evap,i_ = 7.9348 × 10^−10^ mol·cm^−2^·s^−1^, D_SC,i_ = 6.9499 × 10^−10^ cm^2^·s^−1^, and P_SCw,i_ = 5.9352. (**a**) Spatial solute distribution across all domains, including the evaporation region, vehicle, skin layers, and receptor fluid. (**b**) Solute distribution in the receptor fluid.

Figure 2 shows that for 4-Tolunitrile, use of the FSG-based evaporation rate overpredicts evaporative loss and substantially underpredicts cumulative uptake in the receptor fluid (RF). Specifically, temporal profiles indicate the vehicle concentration drops slowly; the stratum corneum (SC) concentration reaches a peak of 10.2747% at 0.0325 h before declining; and the RF exhibits a short lag phase before increasing steadily to 2.4772% at 24 h and reaching a plateau. Further baseline diagnostics are detailed in Supplementary Figure S1. Figure S1a shows that the model maintained good mass conservation throughout the simulation period. Figure S1b displays the solute mass distribution among the vehicle, evaporated fraction, skin, and RF under the low-volatility assumption, reflecting excessive evaporation and insufficient skin/RF permeation. Figure S1c records that the activity gradient—specifically the initial solute activity (0.1333)/solvent activity (0.8667)—changed too rapidly, inconsistent with the experimentally observed retention of solute.

**Figure 3:**
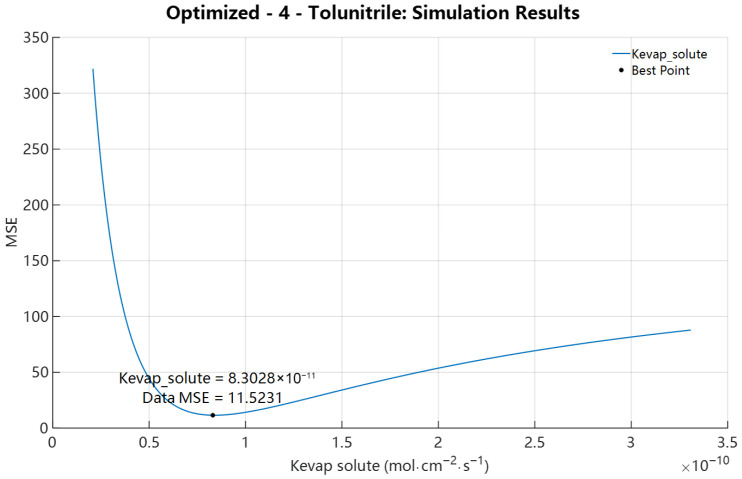
Single-parameter K_evap,i_ sensitivity analysis of 4-Tolunitrile with D_SC,i_ = 6.9499 × 10^−10^ cm^2^·s^−1^ and P_SCw,i_ = 5.9352. The black dot denotes the point yielding the minimum MSE among all sampled values, and the corresponding parameter value is annotated directly adjacent to the marker.

To assess the sensitivity of model predictions to the evaporation rate constant, we performed a 150-point one-dimensional scan of K_evap,i_ over a range spanning two orders of magnitude centred on the FSG estimate. The minimum MSE occurs near K_evap,i_ = 8.3028 × 10^−11^ mol·cm^−2^·s^−1^ (MSE = 11.5231), as shown in Figure 3. Subsequent single-parameter optimization converged to K_evap,i_ = 8.3929 × 10^−11^ mol·cm^−2^·s^−1^ from the initial calculated value 7.9348 × 10^−10^ mol·cm^−2^·s^−1^ (MSE = 11.5124).

**Figure 4:**
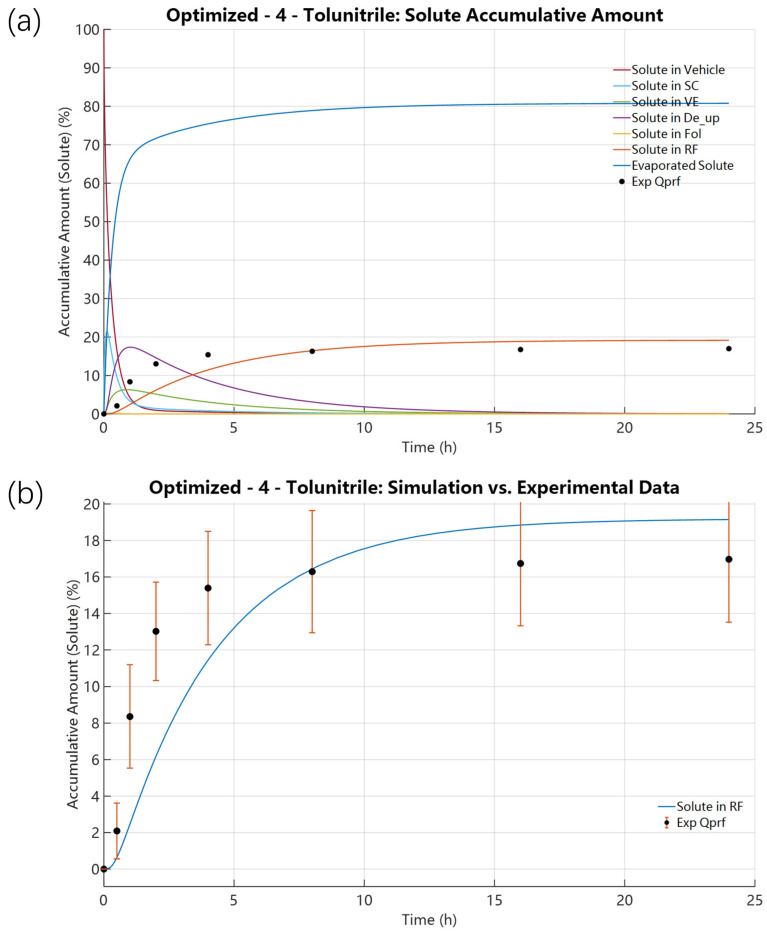
Solute distributions under preliminary K_evap,i_ optimization of 4-Tolunitrile: K_evap,i_ = 8.3929 × 10^−11^ mol·cm^−2^·s^−1^. (D_SC,i_ = 6.9499 × 10^−10^ cm^2^·s^−1^ and P_SCw,i_ = 5.9352). (**a**) Spatial solute distribution across all domains, including the evaporation region, vehicle, skin layers, and receptor fluid. (**b**) Solute distribution in the receptor fluid.

The optimized results reproduced the experimentally observed temporal behaviour of evaporation and transdermal permeation, with the solute depletion process significantly decelerated to match the experimental data (Figure 4). Specifically, the SC concentration peak was predicted at 21.3957% (t = 0.1173 h); the underlying dermal layers VE and De showed peaks at 6.3141% (t = 0.8935 h) and 17.3692% (t = 1.0186 h), respectively, consistent with enhanced retention in skin layers due to decreased volatility. RF accumulation grew steadily after an initial lag, with the final 24 h accumulation at 19.1471%, which compares well with the experimental value of 16.97% (within the error bars). The optimized diagnostic plots (Supplementary Figure S2) further confirm model improvement: Figure S2a reconfirms mass conservation, Figure S2b displays the corrected mass distribution with a substantial decrease in the evaporated fraction and increase in skin and RF permeation, and Figure S2c shows that the activity gradient change was significantly corrected—the solute/solvent activity ratio (initial 0.1333/0.8667) equilibrates more gradually, matching experimental observations of slower solute depletion.

**Figure 5:**
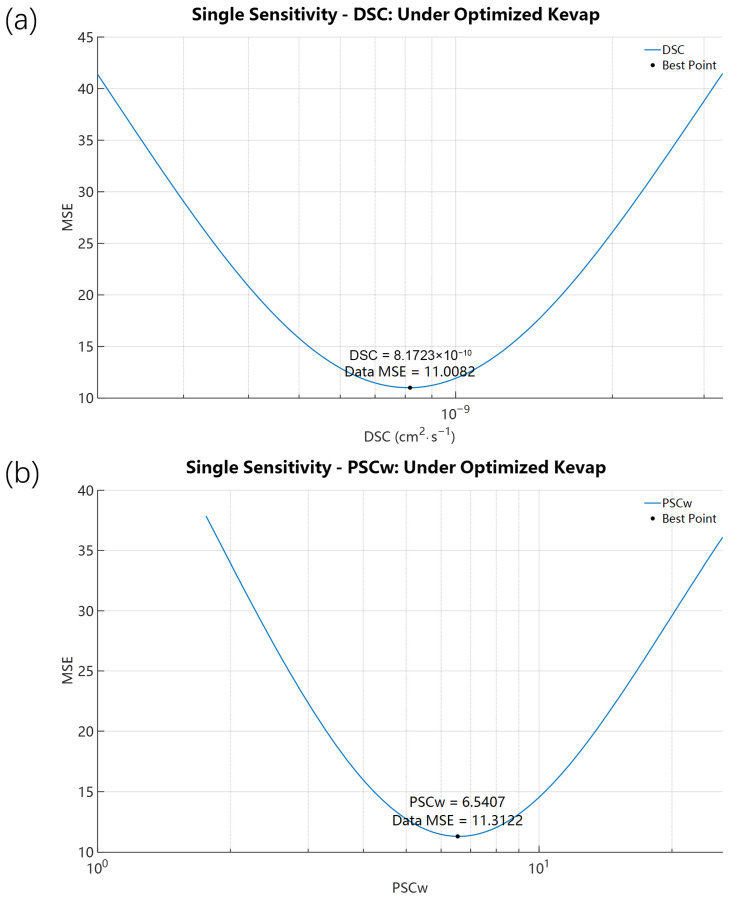
Single-parameter sensitivity analysis of 4-Tolunitrile with K_evap,i_ fixed at its optimized value (8.3929 × 10^−11^ mol·cm^−2^·s^−1^). Panels show MSE responses when scanning (**a**) D_SC,i_ (P_SCw,i_ fixed at 5.9352) and (**b**) P_SCw,i_ (D_SC,i_ fixed at 6.9499 × 10^−10^ cm^2^·s^−1^).

With the optimized K_evap,i_ = 8.3929 × 10^−11^ mol·cm^−2^·s^−1^ held fixed, we further investigated the effects of SC diffusivity (D_SC,i_) and the partition coefficient (P_SCw,i_). Initial values for these parameters were derived from the permeability coefficient k_p,i_ calculated using the QSPR regression equations of Potts and Guy [39]. Scan bounds were set to 0.2–5× the initial values to capture biological variability across skin samples. The single-parameter scan results (Figure 5) indicate that, despite the existence of optimal values for these internal parameters, the resulting improvement in RF prediction was limited. Specifically, from an initial D_SC,i_ = 6.9499 × 10^−10^ cm^2^·s^−1^, the single-parameter optimum converged to 8.1723 × 10^−10^ cm^2^·s^−1^, slightly reducing MSE from 11.5124 to 11.0082. Similarly, the optimum for P_SCw,i_ converged to 6.5407 (near the initial 5.9352), yielding MSE = 11.3122. While optimization of these internal parameters slightly reduced MSE, the substantial MSE reduction achieved by the K_evap,i_ optimization renders the marginal gain from internal-parameter tuning small. This further supports that for highly volatile compounds like 4-Tolunitrile, evaporation is significant in the overall transport process. Importantly, the dominance of evaporation predicted by the model is directly aligned with the intrinsic volatility of both compounds, explaining why calibration of the evaporation rate constant (K_evap,i_) leads to the largest improvement in model performance. In contrast, further sensitivity scans and optimization of stratum corneum transport parameters (D_SC,i_ and P_SCw,i_) produced only marginal reductions in MSE.

**Figure 6:**
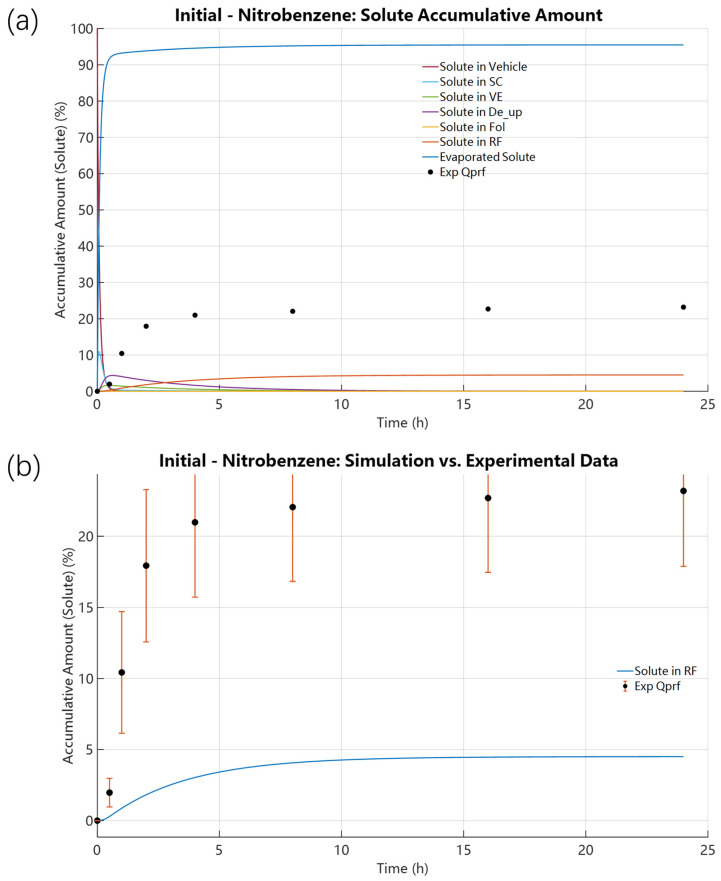
Solute distributions under the initial calculated value of Nitrobenzene: K_evap,i_ = 6.6480 × 10^−10^ mol·cm^−2^·s^−1^, D_SC,i_ = 5.2677 × 10^−10^ cm^2^·s^−1^, and P_SCw,i_ = 4.8645. (**a**) Spatial solute distribution across all domains, including the evaporation region, vehicle, skin layers, and receptor fluid. (**b**) Solute distribution in the receptor fluid.

**Figure 7:**
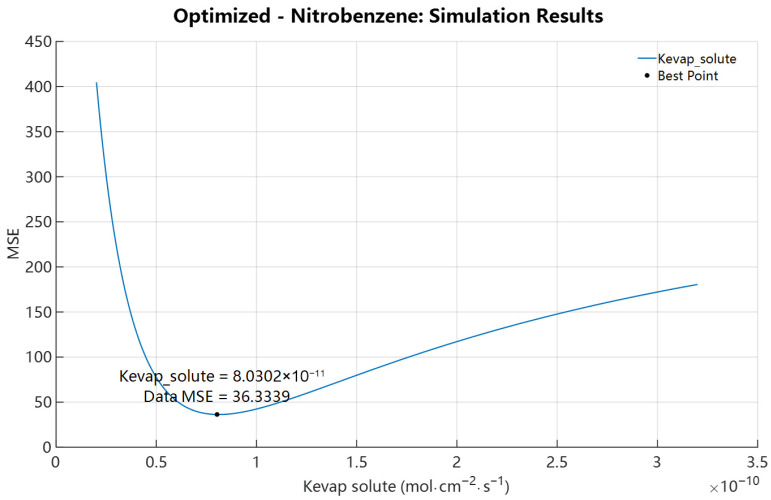
Single-parameter K_evap,i_ sensitivity analysis of Nitrobenzene with D_SC,i_ = 5.2677 × 10^−10^ cm^2^·s^−1^ and P_SCw,i_ = 4.8645. The black dot denotes the point yielding the minimum MSE among all sampled values, and the corresponding parameter value is annotated directly adjacent to the marker.

**Figure 8:**
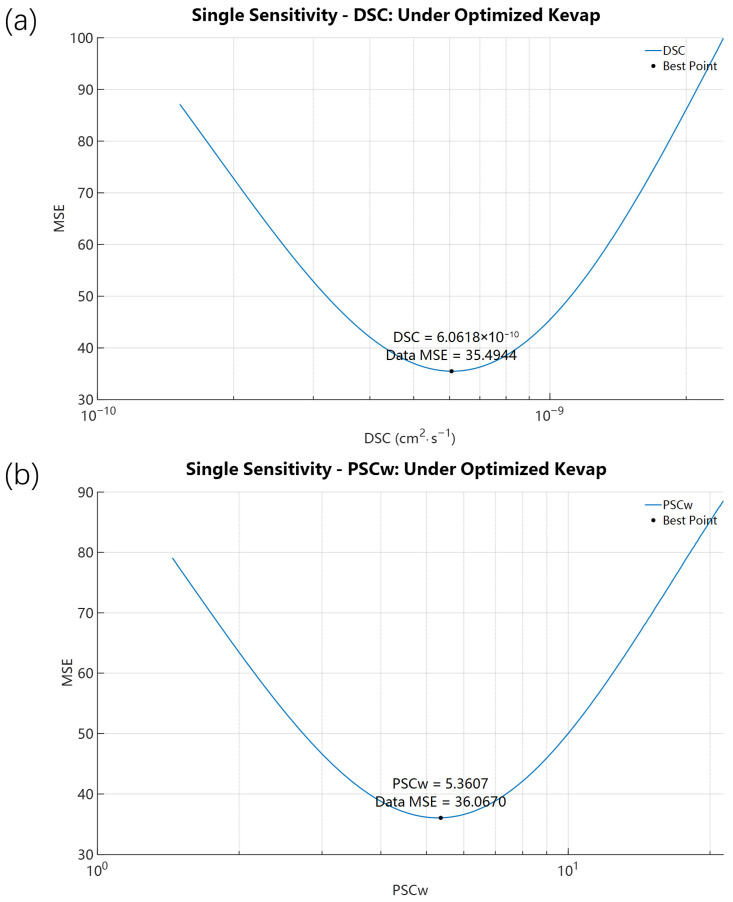
Single-parameter sensitivity analysis of Nitrobenzene with K_evap,i_ fixed at its optimized value (8.1174 × 10^−11^ mol·cm^−2^·s^−1^). Panels show MSE responses when scanning: (**a**) D_SC,i_ (P_SCw,i_ fixed at 4.8645) and (**b**) P_SCw,i_ (D_SC,i_ fixed at 5.2677 × 10^−10^ cm^2^·s^−1^).

Following validation and calibration for 4-Tolunitrile, we applied the same analysis to Nitrobenzene to test model robustness for compounds with different volatility. Compared to 4-Tolunitrile, Nitrobenzene showed higher volatility in open-dish experiments (89% vs. 80.40%) but lower cumulative mass loss in IVPT experiments (66.87% vs. 80.40%). Initial forward simulations for Nitrobenzene, using its FSG theoretical initial K_evap,i_ = 6.6480 × 10^−10^ mol·cm^−2^·s^−1^, resulted in a 24 h RF accumulation of only 4.5027% with the SC solute concentration peaking at 10.9796% at 0.0700 h (Figure 6). These results mirror those for 4-Tolunitrile: the FSG-based initial evaporation rate overpredicted evaporation and underpredicted permeation (predicted 4.5027% permeated vs. experimental 23.19%). The single-parameter scan of K_evap,i_ (Figure 7) produced an optimized K_evap,i_ = 8.1174 × 10^−11^ mol·cm^−2^·s^−1^, approximately one-eighth of the initial value. Compared with the SE-derived initial K_SEevap,i_ = 3.9837 × 10^−12^ mol·cm^−2^·s^−1^, the optimized K_evap,i_ is almost 20.38 times higher. With the optimized value, predicted SC peak rose to 19.5214% (t = 0.1865 h) and RF accumulation to 27.6147% at 24 h, in reasonable agreement with the experimental RF of 23.19%. The results in Figure 8 further demonstrate that, following the initial optimization of K_evap,i_, although further refinement of skin parameters yielded improved outcomes, the resulting enhancement in predicting RF accumulation remained limited. A comparable trend was consistently identified in the case of 4-Tolunitrile.

Table 2 reports the accumulated percentages of the applied dose at 24 h. Calibration of K_evap,i_ alone brings the modelled accumulation distributions for the exposure-relevant parts of the receptor fluid (RF), as well as the evaporation loss (Evap), into close agreement with the experiment. For 4-Tolunitrile, the optimized model predicts RF = 19.15% and Evap = 80.78% (experimental RF = 16.97% and Evap = 80.69%); for Nitrobenzene, the optimized model predicts RF = 27.61% and Evap = 72.28% (experimental RF = 23.19% and Evap = 66.89%). The model systematically underpredicts residual mass in the vehicle and, to a lesser extent, in the skin layers; both have significantly low percentages.

**Figure 9:**
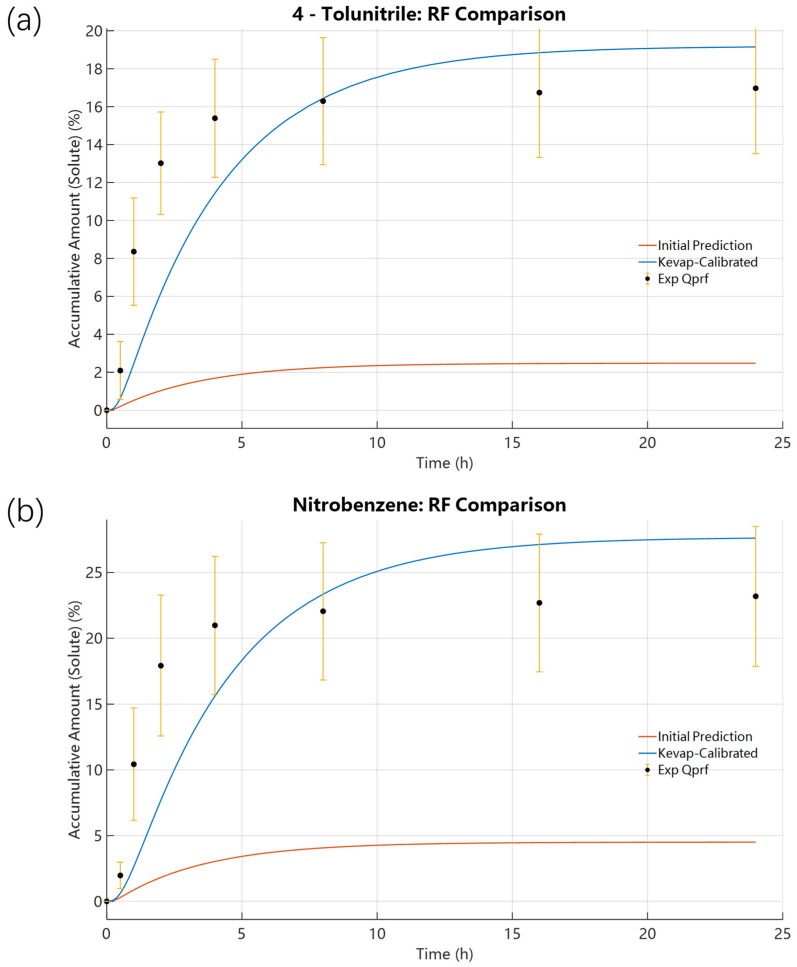
Comparison of RF concentration profiles generated under two parameter sets for 4-Tolunitrile and Nitrobenzene. (**a**) 4-Tolunitrile: (i) The initial prediction (K_evap,i_ = 7.9348 × 10^−10^ mol·cm^−2^·s^−1^, D_SC,i_ = 6.9499 × 10^−10^ cm^2^·s^−1^, and P_SCw,i_ = 5.9352); (ii) the K_evap,i_-calibrated case (K_evap,i_ = 8.3929 × 10^−11^ mol·cm^−2^·s^−1^, D_SC,i_ = 6.9499 × 10^−10^ cm^2^·s^−1^, and P_SCw,i_ = 5.9352). (**b**) Nitrobenzene: (i) The initial prediction (K_evap,i_ = 6.6480 × 10^−10^ mol·cm^−2^·s^−1^, D_SC,i_ = 5.2677 × 10^−10^ cm^2^·s^−1^, and P_SCw,i_ = 4.8645); (ii) the K_evap,i_-calibrated case (K_evap,i_ = 8.1174 × 10^−11^ mol·cm^−2^·s^−1^, D_SC,i_ = 5.2677 × 10^−10^ cm^2^·s^−1^, and P_SCw,i_ = 4.8645).

**Figure 10:**
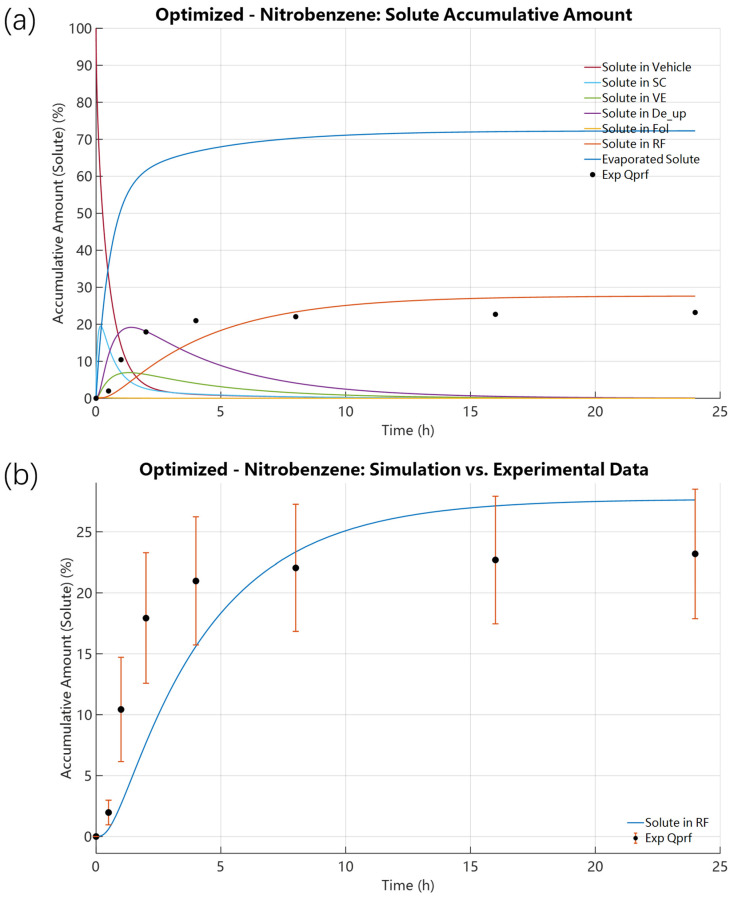
Solute distributions under preliminary K_evap,i_ optimization of Nitrobenzene: K_evap,i_ = 8.1174 × 10^−11^ mol·cm^−2^·s^−1^. (D_SC,i_ = 5.2677 × 10^−10^ cm^2^·s^−1^ and P_SCw,i_ = 4.8645). (**a**) Spatial solute distribution across all domains, including the evaporation region, vehicle, skin layers, and receptor fluid. (**b**) Solute distribution in the receptor fluid.

Note that for both compounds, optimized K_evap,i_ values are roughly one order of magnitude lower than the FSG estimates (≈1/10 for 4-Tolunitrile and ≈1/8 for Nitrobenzene), yet more than 20× higher than SE-based estimates. Figure 9 compares the initial model predictions with the optimized results for the two investigated compounds. It can be observed that the optimized results show good agreement with the experimental measurements. The quantitative results and diagnostic plots are reported in Figure 2, Figure 3, Figure 4, Figure 5, Figure 6, Figure 7, Figure 8, Figure 9 and Figure 10 and Supplementary Figures S1 and S2.

## 5. Discussion

The results demonstrate that accurate representation of gas-phase evaporation is critical for reproducing IVPT permeation kinetics of highly volatile solutes. Using uncalibrated FSG-derived evaporation constants produced excessive evaporative loss and underpredicted RF uptake for both compounds. Optimization of the lumped evaporation coefficient K_evap,i_ substantially improved agreement with experiment, indicating that while the FSG estimate is physically motivated, it overestimates effective evaporation under the tested IVPT conditions.

Several factors may explain the systematic reduction in optimized K_evap,i_ relative to FSG predictions. First, the effective evaporation process in the IVPT donor chamber may include additional resistances not resolved by the gas-phase kinetic estimate, for example, liquid–air interfacial transport effects or near-surface microenvironments. Second, the donor-chamber geometry and limited ventilation can reduce convective mass transfer so that a free-air kinetic-theory estimate overpredicts mass loss. In our implementation, the volatile diffusion path length was set to h = 2 cm to mimic the typical donor-chamber geometry (i.e., the distance from the formulation surface to the surrounding ambient environment) in the IVPT experiments; this is a pragmatic approximation in the absence of detailed local airflow characterization.

The observation that optimized K_evap,i_ values are substantially larger than SE-based estimates confirms that SE (a liquid-phase relation) is inappropriate for gas-phase evaporation modelling. Conversely, the need to reduce the FSG-derived values highlights that kinetic-theory-based estimates should be interpreted cautiously and treated as starting points for calibration under confined or quiescent experimental conditions.

Sensitivity analysis indicates that once K_evap,i_ is optimized, further tuning of internal skin parameters such as D_SC,i_ and P_SCw,i_ yields only marginal improvements in RF prediction. This supports the conclusion that, for highly volatile solutes under finite-dose IVPT, gas-phase losses dominate the mass balance and primarily determine cumulative permeation; thus, prioritizing characterization of evaporation-related parameters and experimental boundary conditions (donor geometry, diffusion path length, and ventilation) will more effectively improve model–experiment agreement than exhaustive recalibration of internal skin transport parameters.

The layerwise comparison in Table 2 clarifies the scope and limits of the present model. First, the calibration of a single lumped gas-phase parameter K_evap,i_ predicts well both RF and evaporative loss demonstrates that the model is valid by incorporating the evaporation as the main rate-controlling process for transdermal permeation of volatile solutes. Second, the model systematically underpredicts the small residual percentages reported in the vehicle and certain skin sub-compartments. This can be either that the percentages in the skin and vehicle are insignificant for model calibration or a shortcoming of the current model. The inclusion of additional mechanistic terms, such as solvent evaporation or concurrent permeation of both volatile solute and solvents, is necessary in future refinements.

The results can also be compared with recent compartmental modelling work. Fisher et al. developed a compartmental model of transdermal permeation that also treats volatile evaporation as affected by air velocity and exposed area. They highlighted that vehicle dry-down and stratum corneum permeability were dominant sensitivities and reported broadly reasonable predictions within an order of magnitude error with experimental data [40].

Methodologically, our findings imply two practical recommendations for future IVPT and modelling studies: (i) report donor-chamber geometry and any ventilation/local airflow conditions explicitly; and (ii) where direct airflow/evaporation measurements are not available, treat kinetic-theory estimates of K_evap,i_ as initial values to be calibrated rather than as fixed predictions.

## 6. Conclusions

The proposed mechanistic modelling framework for coupling evaporation and transdermal permeation of volatile compounds is robust for modelling IVPT data of finite-dose topical formulations. Methodologically, the framework couples Fickian gas-phase evaporation with multilayer transdermal diffusion, both driven by the activity of volatiles in the vehicle. The dynamic change in volatile activity coefficients is computed on-the-fly using UNIFAC within a hybrid MATLAB-Python architecture. This implementation permits mechanistic treatment of non-ideal multicomponent vehicles and transient changes in formulation composition during both evaporation and transdermal permeation. It provides a generic platform for further research on co-solvent and emulsion systems.

Initial validation of the model with published IVPT data showed the optimized evaporation coefficient K_evap,i_ is reduced from the Fuller–Schettler–Giddings (FSG) based initial estimates by roughly an order of magnitude (4-Tolunitrile: from 7.9348 × 10^−10^ to 8.3929 × 10^−11^ mol·cm^−2^·s^−1^; Nitrobenzene: from 6. 6480 × 10^−10^ to 8.1174 × 10^−11^ mol·cm^−2^·s^−1^). The predicted accumulative amounts at 24 h for both evaporative loss and receptor fluid agree well with experimental data. For highly volatile compounds of 4-Tolunitrile and Nitrobenzene, gas-phase evaporation constitutes the dominant mass-loss, limiting the cumulative amount permeated to the receptor fluid.

## 7. Limitations and Future Work

The present model focuses on the development of a modelling framework that couples evaporative loss with transdermal permeation of volatiles. Although the approach is generic and applies to both volatile solutes and solvents from multi-component complex formulations, initial validation is limited to the IVPT data of two volatiles in aqueous solution. Looking forward, three complementary developments are necessary. First, extend the validation of the model to explicitly account for the evaporation of both solvent and permeants. Second, exploit the existing UNIFAC implementation to compute on-the-fly the activity coefficients of volatiles in non-ideal solutions consisting of multi-components such as co-solvent systems. Third, modelling concurrent permeation of both solute and solvents may be necessary to fully elucidate the formulation effect on transdermal permeation.

## Figures and Tables

**Table 1:** Physicochemical properties of chemicals and the properties calculated using QSPR formulas and the evaporation module. (Experimental temperature T was 305.15 K. The Boltzmann constant used was *k_b_* = 1.3806 × 10^−23^ J·K^−1^. For SE estimates, the air dynamic viscosity at the experimental temperature was taken as η_air_ = 1.8710 × 10^−5^ Pa·s; water dynamic viscosity provided in input was η_water_ = 7.6441 × 10^−4^ Pa·s. The evaporation path length used in the K_evap,i_ calculations was h = 2 cm. The universal gas constant used in conversions is R = 8.314462618 J·mol^−1^·K^−1^).

Properties (Unit)	Symbol	4-Tolunitrile	Nitrobenzene
Molecular weight (g·mol^−1^)	MW	117.15	123.11
Octanol/water partition (-)	LogP	2.09	1.85
Molecular/Stokes radius (m)	r_s_	2.9401 × 10^−10^	2.9891 × 10^−10^
Vapour pressure (Pa)	P_v_	41.7298	32.6639
Vehicle diffusion coefficient (cm^2^·s^−1^)	D_veh_	9.9378 × 10^−6^	9.7748 × 10^−6^
SC diffusion coefficient (cm^2^·s^−1^)	D_SC_	6.9499 × 10^−10^	5.2677 × 10^−10^
Follicle/water diffusion coefficient (cm^2^·s^−1^)	D_Fol,water_	9.9378 × 10^−6^	9.7748 × 10^−6^
VE/dermis diffusion coefficient (cm^2^·s^−1^)	D_vede_	1.0176 × 10^−6^	1.1790 × 10^−6^
Octanol/water partition coefficient (-)	P_ow_	123.0269	70.7946
SC lipid/water partition coefficient (-)	P_lw_	24.9082	17.0115
SC keratin/water partition coefficient (-)	P_prw_	25.5782	21.5511
SC/water partition coefficient (-)	P_SCw_	5.9352	4.8645
VE/dermis–water partition coefficient (-)	P_vedew_	2.1503	1.7966
Permeability coefficient (Potts & Guy) (cm·s^−1^)	k_p_	2.9464 × 10^−6^	1.8303 × 10^−6^
Fraction unbound (-)	f_u_	0.1809	0.2190
Fraction non-ionized (-)	f_non_	1	1
FSG: Gas-phase diffusivity coefficient (cm^2^·s^−1^)	D_evap_	9.6481 × 10^−2^	1.0327 × 10^−1^
FSG: Mass-transfer coefficient (mol·cm^−2^·s^−1^)	K_evap_	7.9348 × 10^−10^	6.6480 × 10^−10^
SE: Gas-phase diffusivity coefficient (cm^2^·s^−1^)	D_SEevap_	4.0501 × 10^−4^	3.9837 × 10^−4^
SE: Mass-transfer coefficient (mol·cm^−2^·s^−1^)	K_SEevap_	3.3309 × 10^−12^	2.5645 × 10^−12^

**Table 2:** Experimental vs. model: predicted distribution (%) at 24 h.

Compound	Compartment	Experimental Results ^a^ (%)	Initial Results ^b^ (%)	Optimized Results ^c^ (%)
4-Tolunitrile	Vehicle	2.26	1.5019 × 10^−12^	0.002709
	SC	0.04	0.0001641	0.004525
	VE	0.03	0.000835	0.01724
	De	0.01	0.002387	0.04904
	RF	16.97	2.477	19.15
	Evaporation	80.69	97.52	80.78
Nitrobenzene	Vehicle	7.5	0.0001088	0.006007
	SC	1.71	0.0002988	0.006752
	VE	0.49	0.001369	0.02407
	De	0.22	0.003888	0.06814
	RF	23.19	4.503	27.61
	Evaporation	66.89	95.49	72.28

^a^ Experimental results are taken from the Supporting Information of Hewitt et al. [29]; ^b^ Initial results denote forward simulations using the FSG-based initial estimate of K_evap,i_. ^c^ Optimized results denote model predictions after calibration of K_evap,i_.

## Data Availability

The original contributions presented in this study are included in the article/Supplementary Material. Further inquiries can be directed to the corresponding author.

## Supplementary Materials

The following supporting information can be downloaded at: https://www.mdpi.com/article/10.3390/pharmaceutics18020221/s1, Section S1: Model Mathematical and Numerical Derivations; Section S2: Physicochemical Parameter Estimation; Section S3: Parameter Optimization and Sensitivity Analysis Methodology; Figure S1: Solvent–solute behaviour and vehicle evolution under the initial calculated value of 4-Tolunitrile (K_evap,i_ = 7.9348 × 10^−10^ mol·cm^−2^·s^−1^, D_SC,i_ = 6.9499 × 10^−10^ cm^2^·s^−1^, P_SCw,i_ = 5.9352). (a) Solute and solvent mass conservation trajectories evaluated under the initial calculated value. (b) Spatial distributions of solute and solvent concentrations together with the vehicle height profile across the evaporation region, vehicle layer, skin domains, and receptor fluid. (c) Temporal evolution of solute and solvent activities within the vehicle under the same initial parameter set; Figure S2: Solvent–solute behaviour and vehicle evolution under preliminary K_evap,i_ optimization of 4-Tolunitrile (K_evap,i_ = 8.3929 × 10^−11^ mol·cm^−2^·s^−1^, D_SC,i_ = 6.9499 × 10^−10^ cm^2^·s^−1^, P_SCw,i_ = 5.9352). (a) Solute and solvent mass conservation trajectories evaluated under the initial calculated value. (b) Spatial distributions of solute and solvent concentrations together with the vehicle height profile across the evaporation region, vehicle layer, skin domains, and receptor fluid. (c) Temporal evolution of solute and solvent activities within the vehicle under the same initial parameter set; Figure S3: Solvent–solute behaviour and vehicle evolution under the initial calculated value of Nitrobenzene (K_evap,i_ = 6.6480 × 10^−10^ mol·cm^−2^·s^−1^, D_SC,i_ = 5.2677 × 10^−10^ cm^2^·s^−1^, P_SCw,i_ = 4.8645). (a) Solute and solvent mass conservation trajectories evaluated under the initial calculated value. (b) Spatial distributions of solute and solvent concentrations together with the vehicle height profile across the evaporation region, vehicle layer, skin domains, and receptor fluid. (c) Temporal evolution of solute and solvent activities within the vehicle under the same initial parameter set; Figure S4: Solvent–solute behaviour and vehicle evolution under preliminary K_evap,i_ optimization of Nitrobenzene (K_evap,i_ = 8.1174 × 10^−11^ mol·cm^−2^·s^−1^, D_SC,i_ = 5.2677 × 10^−10^ cm^2^·s^−1^, P_SCw,i_ = 4.8645). (a) Solute and solvent mass conservation trajectories evaluated under the initial calculated value. (b) Spatial distributions of solute and solvent concentrations together with the vehicle height profile across the evaporation region, vehicle layer, skin domains, and receptor fluid. (c) Temporal evolution of solute and solvent activities within the vehicle under the same initial parameter set.

## Abbreviations

IVPT	in vitro permeation test
QSPR	quantitative structure property relationships
PBPK	physiologically based pharmacokinetics
NPts	number of points
SE	Stokes–Einstein
FSG	Fuller–Schettler–Giddings
MW	molecular weight
MSE	mean-square error
RSS	residual sum of squares
SC	stratum corneum
VE	viable epidermal
De	dermis
Fol	hair follicular
RF	receptor fluid
PBS	phosphate-buffered saline
