# Supplementary material for: Modelling Transdermal Permeation of Volatiles from Complex Product Formulations
Source: Pharmaceutics. 2026 Feb 9;18(2):221. doi: 10.3390/pharmaceutics18020221 (PMC12943934; doi:10.3390/pharmaceutics18020221)
Supplement: Supplementary file 1 [file pharmaceutics-18-00221-s001.zip › pharmaceutics-4078840-supplementary.pdf]

Title

Modelling transdermal permeation of volatiles from complex product formulations

Author

Zhihao Zhong[a,b], Guoping Lian[a], Tao Chen[a], Yuan Yu[b]

a Department of Chemical and Process Engineering, University of Surrey, Guildford GU2 7XH,

b College of Safety Science and Engineering, Nanjing Tech University, Nanjing 211816, China

* Corresponding author at: Guoping Lian g.lian@surrey.ac.uk

Supporting Information

Section S1. Model Mathematical and Numerical Derivations

The simulation model is based on the Finite Difference numerical procedure of previous work. The skin layers are discretized into a finite number of grids. The governing equation, based on the laws of mass conservation for each grid element i after spatial discretization (the coupled ODE system), is defined as:

$$V_i \frac{dC_i}{dt} = -\sum_j J_{ij}$$

Where V_i is the volume of grid i, C_i is the concentration in skin grid i, and the right-hand side sums the fluxes of all grids adjacent to grid i. This system of ODEs is derived from the overall Finite Difference Method (FDM) spatial discretization scheme.

The mass transfer flux (q_{ij}) between two adjacent layers or meshes i and j is calculated using the resistance-based formulation of interfacial mass transfer:

$$J_{ij} = \frac{A}{\frac{\delta_i}{D_i} + \frac{P_{ij}\delta_j}{D_j}}\left(C_i - K_{ij}C_j\right)$$

Where J_{ij} is the flux from grid i to grid j (in mass/time, e.g., kg/s or mol/s), A is the interface area, δ_i and δ_j are the respective lengths of the diffusion pathway, D_i and D_j are the corresponding diffusion coefficients, P_{ij} is the partition coefficient of the permeant between grid i and grid j, and C_i and C_j are the concentrations of grid i and grid j, respectively.

By the way, the calculation of P_{ij} is present here:

$$P_{ij} = \frac{P_{iw}}{P_{jw}}$$

Where the P_{iw} is the partition coefficient between phase i and water, the P_{jw} is the partition coefficient between phase j and water, for example, the partition coefficient between SC and vehicle can be present as:

$$P_{SC/veh} = \frac{P_{SC/w}}{P_{veh/w}}$$

The system is discretized using the following node counts (M):

$$M_v = 1$$

$$M_{SC} = 20$$

$$M_{VE} = 20$$

$$M_{De_{up}} = 20$$

$$M_{Fol} = 60$$

The mass balance for the vehicle incorporates the evaporative flux J_{evap} (Eq. (11) in main text) and

transdermal penetration:

$$\frac{dM_{solute}}{dt} = -J_{evap} - J_{out\_SC} - J_{out\_Fol}$$

The first SC and Follicle nodes are explicitly coupled to the Vehicle mass balance. The evaporative flux J_{evap} is calculated from the real-time activity coefficient (γ) of the solute in the vehicle, which is dynamically calculated using the thermodynamic model UNIFAC.

The mass balance ODE for a skin node i adjacent to a follicle node j includes a lateral flux term (J_{lat}), for the shunt pathway to be integrated:

$$\frac{dM_i}{dt} = J_{in} - J_{out} \pm J_{lat}$$

The RF mass conservation is given by:

$$\frac{dM_{RF}}{dt} = J_{SC\_to\_RF} + J_{Fol\_to\_RF}$$

The model assumes a perfect sink condition where the RF concentration is negligible relative to the bottom skin layer concentration, simplifying the transport across the bottom interface.

This coupled ODE system is integrated over time using the stiff solver ode15s in MATLAB.

Section S2. Physicochemical Parameter Estimation

The parameters required for transport and partitioning calculations are derived from established empirical or physically based formulae, detailed as follows:

Permeant Radius:

The molecular radius of the permeant (r_s in Å) is calculated from the molecular weight (MW in g·mol^{-1}):

$$r_s = \sqrt[3]{3/4\pi \times 0.9087MW}$$

Vehicle Properties:

Partition Coefficients (P_{vw}): The partition coefficient of the permeant between vehicle and water can be present by the solubility of permeant in each solvent. For PBS dilute aqueous solution, the vehicle is approximated as the water phase, and the partition coefficient can be represented as:

$$P_{vw} = \frac{S_v}{S_w} = 1$$

Diffusion Coefficient (D_v): The diffusion coefficient (D_v in m^2·s^{-1}) of the permeant in solution (vehicle) is calculated using the Stokes-Einstein equation:

$$D_v = \frac{k_B T}{6\pi\eta r_s}$$

Where k_B is the Boltzmann constant (1.3806×10^{-23} J·K^{-1}), T is the absolute temperature (for 32 C, T = 305 K), eta is the vehicle viscosity (7.64407×10^{-4} Pa·s under 305 K), and r_s is the permeant radius in meters.

Stratum Corneum (SC) Properties:

Partition Coefficients: The partition coefficient of the SC to water (P_{SCw}) is calculated by considering the lipid (ρ_l), keratin (ρ_k), and water (ρ_w) components, using the overall SC density (ρ_{SC}),

$$P_{lw} = \frac{\rho_l}{\rho_w} \times P_{ow}^{0.69}$$

$$P_{prw} = \frac{\rho_k}{\rho_w} \times P_{ow}^{0.31} \times 4.2$$

$$P_{SCw} = (0.1476 \times P_{prw} + 0.0671 \times P_{lw} + 0.7853) \times \frac{\rho_w}{\rho_{SC}}$$

where $\rho_l = 0.9e3$ kg·m^{-3}, $\rho_w = 1e3$ kg·m^{-3}, $\rho_k = 1.37e3$ kg·m^{-3}, $\rho_{SC} = 1.05e3$ kg·m^{-3}.

SC Permeability Coefficient (k_p): Calculated using the Potts and Guy's QSPR equation:

$$k_p = 10^{-6.3\ +\ 0.71 \times LogP\ -\ 0.0061 \times MW}$$

SC Diffusion Coefficient (D_{SC}): Derived from the permeability and partition coefficients, where h_{SC} is the SC thickness:

$$D_{SC} = k_p \times \frac{h_{SC}}{P_{SCw}}$$

Viable Epidermis and Dermis (VE/Dermis) Properties:

The estimation of D_{vede} and P_{vedew} utilizes corrections for protein binding and ionization, based on Yamazaki's research and modified by Chen et al. The protein unbound and nonionized fractions are calculated as: (Note that f_{non} is set to 1 when pKa information is unavailable (pKa not exist).)

$$f_u = \begin{cases} 1 - \dfrac{0.7936 \times \exp(\log P_{ow}) + 0.2239}{0.7936 \times \exp(\log P_{ow}) + 1.2239} & pKa > 7.4\ weak\ acid \\ 1 - \dfrac{0.5578 \times \exp(\log P_{ow}) + 0.0188}{0.5578 \times \exp(\log P_{ow}) + 1.0188} & pKa \leq 7.4\ weak\ base\ or\ neutral \end{cases}$$

$$f_{non} = \begin{cases} \dfrac{1}{1 + 10\text{^}(7.4 - pKa)} & pKa > 7.4\ weak\ acid \\ \dfrac{1}{1 + 10\text{^}(pKa - 7.4)} & pKa < 7.4\ weak\ base \\ 1 & pKa\ not\ exist \end{cases}$$

Binding Factor (BF) and Free Diffusion (D_{free}):

$$BF = 0.65 + \frac{0.32}{f_u} + 0.025 \times f_{non} \times P_{lw}$$

$$D_{free} = 10^{-8.15 - 0.655 \times logMW}$$

Final VE/Dermis Parameters:

$$D_{vede} = \frac{D_{free}}{BF}$$

$$P_{vedew} = 0.7 \times BF$$

Sebum Properties:

These parameters are derived based on the formulation condition (lipid-rich or water-based). The diffusion coefficient of sebum (D_s) for the lipid-rich case is from Yang et al.:

$$D_s = 2.48e - 4 \times e^{-0.42 \times r_s^2}$$

where D_s is in $cm^2 \cdot s^{-1}$ and r_s in Å):

For the water-based case (typical of IVPT using PBS or water-based vehicles), the properties are based on the assumption that the sebum compartment is effectively saturated with water, leading to simple transport properties, a speculation attributed to Sebastia-Saez's work:

$$D_s = D_v$$

$$P_{sw} = 1$$

This assumption reflects the system's shift from lipophilic diffusion to aqueous diffusion under high water content.

Section S3. Parameter Optimization and Sensitivity Analysis Methodology

The methodologies for parameter calibration, single-parameter sensitivity scanning, and two-dimensional joint parameter mapping share a common computational workflow: at each parameter-space point, the model simulates multiple responses, and the Residual Sum of Squares (RSS) across all responses is calculated as the objective function. The RSS is directly used by MATLAB's lsqnonlin (trust-region reflective) solver to determine the optimal parameters. Mean Squared Error (MSE) is calculated only for visualization purposes.

Multi-response Residual Definition

For each parameter point, the model evaluates three types of responses:

1. Evaporation residual (at final simulation time t_{end}):

$$r_1 = \frac{Q_{evap}^{sim}(t_{end}) - y_1^{exp}}{100}$$

2. Receptor fluid cumulative mass residuals (at each experimental time point t_i, i=1…N):

$$r_{2,i} = \frac{Q_{rf}^{sim}(t_i) - Q_{rf}^{exp}(t_i)}{100}, i = 1 \ldots N$$

3. Skin residual (at final simulation time t_{end}):

$$r_3 = \frac{Q_{skin}^{sim}(t_{end}) - y_3^{exp}}{100}$$

The complete residual vector for computing RSS is:

$$r_{current} = \{r_1, r_{2,1}, r_{2,2}, \ldots, r_{2,N}, r_3\}$$

$$RSS = \sum_j r_{current,j}^2$$

For visualization, MSE is defined as:

$$MSE = \frac{RSS}{N_{data}}, N_{data} = N + 2$$

where N is the number of receptor fluid data points, and the extra two points correspond to evaporation and skin.

Application 1: Parameter Calibration (for K_{evap})

The primary optimization focused on determining the best-fit value for K_{evap}. This used a two-step hybrid optimization strategy:

1. Rough Global Search (Grid Search): A 1D grid search (Npts =150) was carried out over the physical bounds $[0.01 \cdot p_0, 100 \cdot p_0]$ for K_{evap}, where p_0 is the initial calculated value. Because p_0 differs between compounds, the actual search intervals were compound-specific: for 4-Tolunitrile, $K_{evap} \in [7.9348 \times 10^{-12}, 7.9348 \times 10^{-8}]$, for Nitrobenzene, $K_{evap} \in [6.6480 \times 10^{-12}, 6.6480 \times 10^{-8}]$, both values are in units of $mol \cdot cm^{-2} \cdot s^{-1}$. The K_{evap} value that produced the minimum residual sum of squares (RSS) within each compound-specific grid was selected as the refined starting point for subsequent optimization.
2. Local Refinement: This starting point was then fed into a high-precision local minimization solver lsqnonlin to converge to the final optimized K_{evap} value by minimizing the RSS.

Application 2: Single-Parameter Sensitivity Scans

Single-parameter scans (Npts =150) were performed with K_{evap} fixed at its optimized value.

For each compound, one parameter (either D_{SC} or P_{SCw}) was varied at a time across its physical range $[0.2 \cdot p_0, 5 \cdot p_0]$ while all other parameters remained fixed. The compound-specific ranges were: for 4-Tolunitrile, $D_{SC} \in [1.3900 \times 10^{-10}, 3.4749 \times 10^{-9}]$ $cm^2 \cdot s^{-1}$ and $P_{SCw} \in [1.1870, 29.6760]$; for

Nitrobenzene, $D_{SC} \in [1.0535\times10{-}10, 2.6338\times10{-}9]$ $cm^2 \cdot s^{-1}$ and $P_{SCw} \in [0.9729, 24.3225]$. At each grid point the RSS and mean squared error (MSE) were evaluated; the resulting MSE profiles were plotted against parameter values to illustrate model sensitivity (see main text).

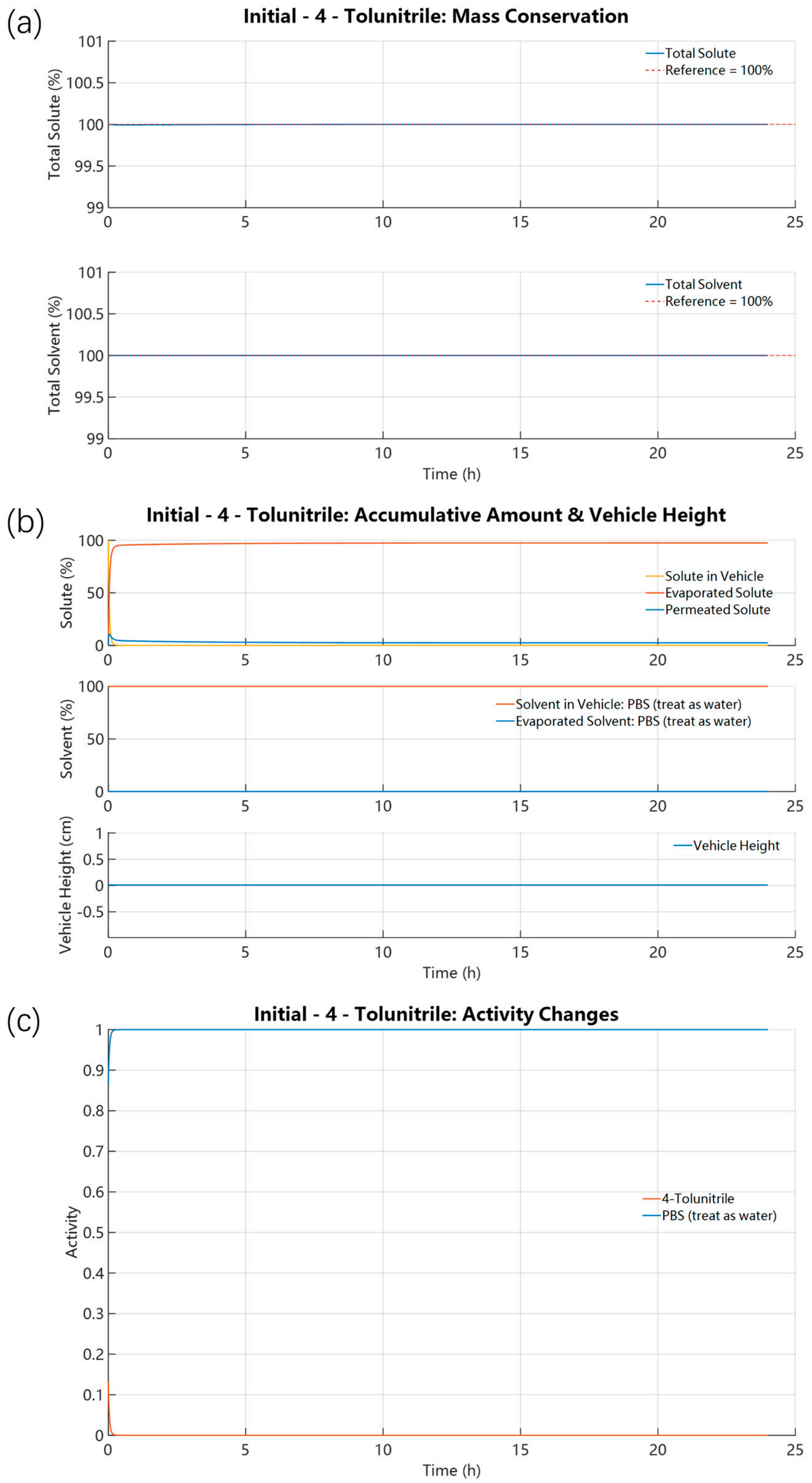

Figure S1 Solvent–solute behaviour and vehicle evolution under the initial calculated value of 4-Tolunitrile ($K_{evap,i} = 7.9348\times10^{-10}$ mol·cm^{-2}·s^{-1}, $D_{SC,i} = 6.9499\times10^{-10}$ cm^{2}·s^{-1}, $P_{SCw,i} = 5.9352$). (a) Solute and solvent mass conservation trajectories evaluated under the initial calculated value. (b) Spatial distributions of solute and solvent concentrations together with the vehicle height profile across the evaporation region, vehicle layer, skin domains, and receptor fluid. (c) Temporal evolution of solute and solvent activities within the vehicle under the same initial parameter set.

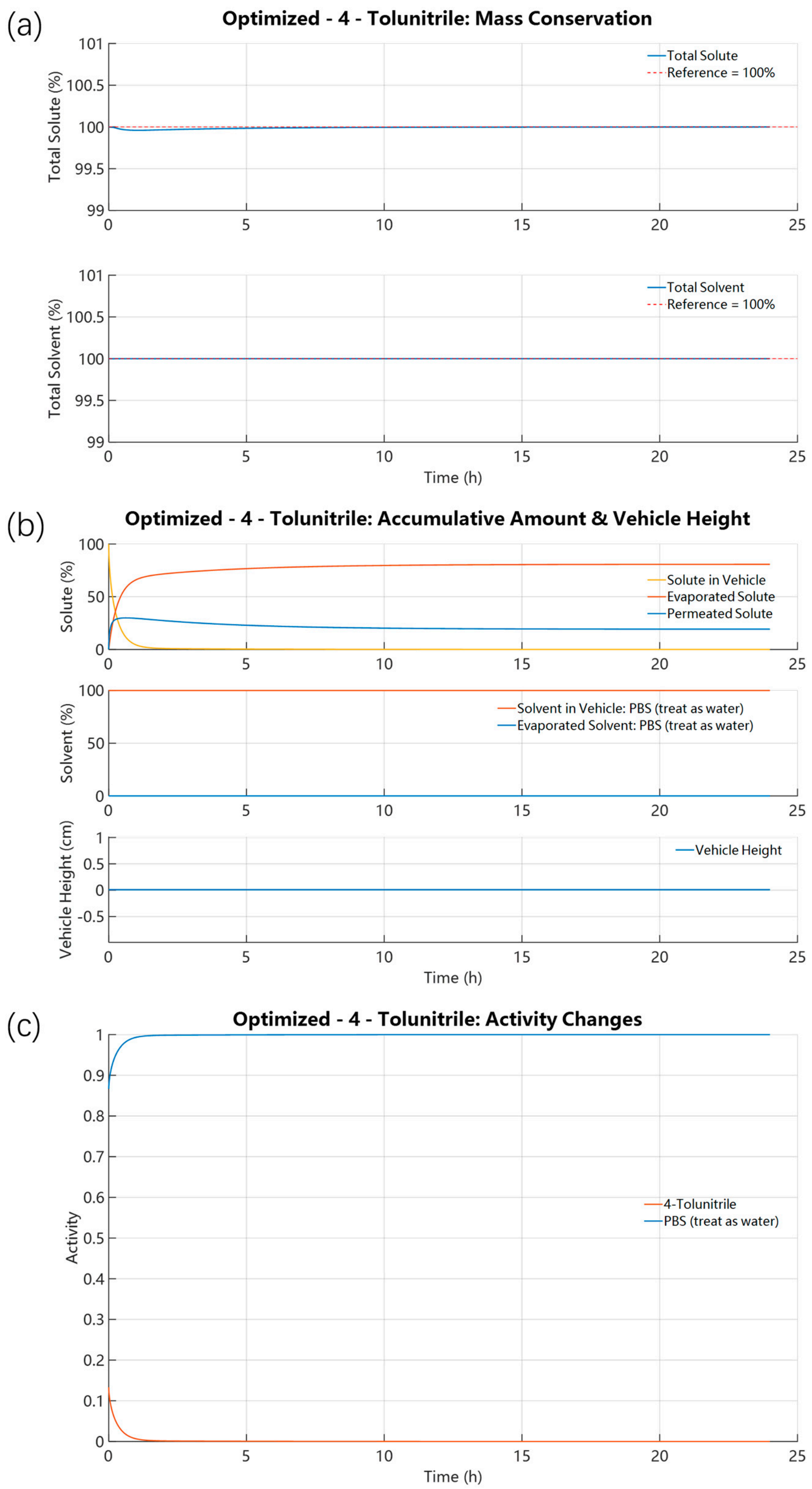

Figure S2 Solvent–solute behaviour and vehicle evolution under preliminary $K_{evap,i}$ optimization of 4-Tolunitrile ($K_{evap,i} = 8.3929\times10^{-11}$ mol·cm^{-2}·s^{-1}, $D_{SC,i} = 6.9499\times10^{-10}$ cm^{2}·s^{-1}, $P_{SCw,i} = 5.9352$). (a) Solute and solvent mass conservation trajectories evaluated under the initial calculated value. (b) Spatial distributions of solute and solvent concentrations together with the vehicle height profile across the evaporation region, vehicle layer, skin domains, and receptor fluid. (c) Temporal evolution of solute and solvent activities within the vehicle under the same initial parameter set.

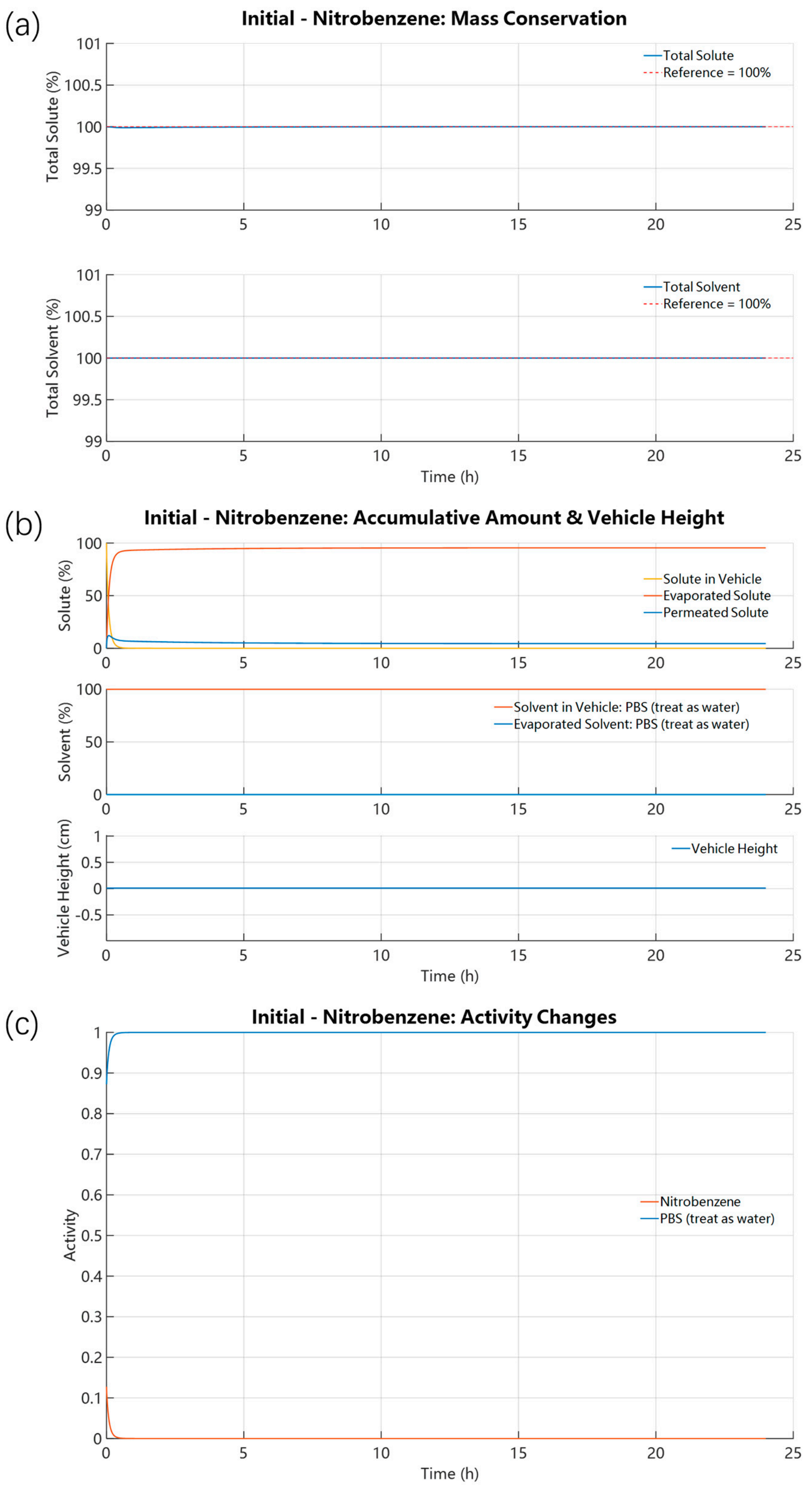

Figure S3 Solvent–solute behaviour and vehicle evolution under the initial calculated value of Nitrobenzene ($K_{evap,i}$ = 6.6480×10^{-10} mol·cm^{-2}·s^{-1}, $D_{SC,i}$ = 5.2677×10^{-10} cm^2·s^{-1}, $P_{SCw,i}$ = 4.8645). (a) Solute and solvent mass conservation trajectories evaluated under the initial calculated value. (b) Spatial distributions of solute and solvent concentrations together with the vehicle height profile across the evaporation region, vehicle layer, skin domains, and receptor fluid. (c) Temporal evolution of solute and solvent activities within the vehicle under the same initial parameter set.

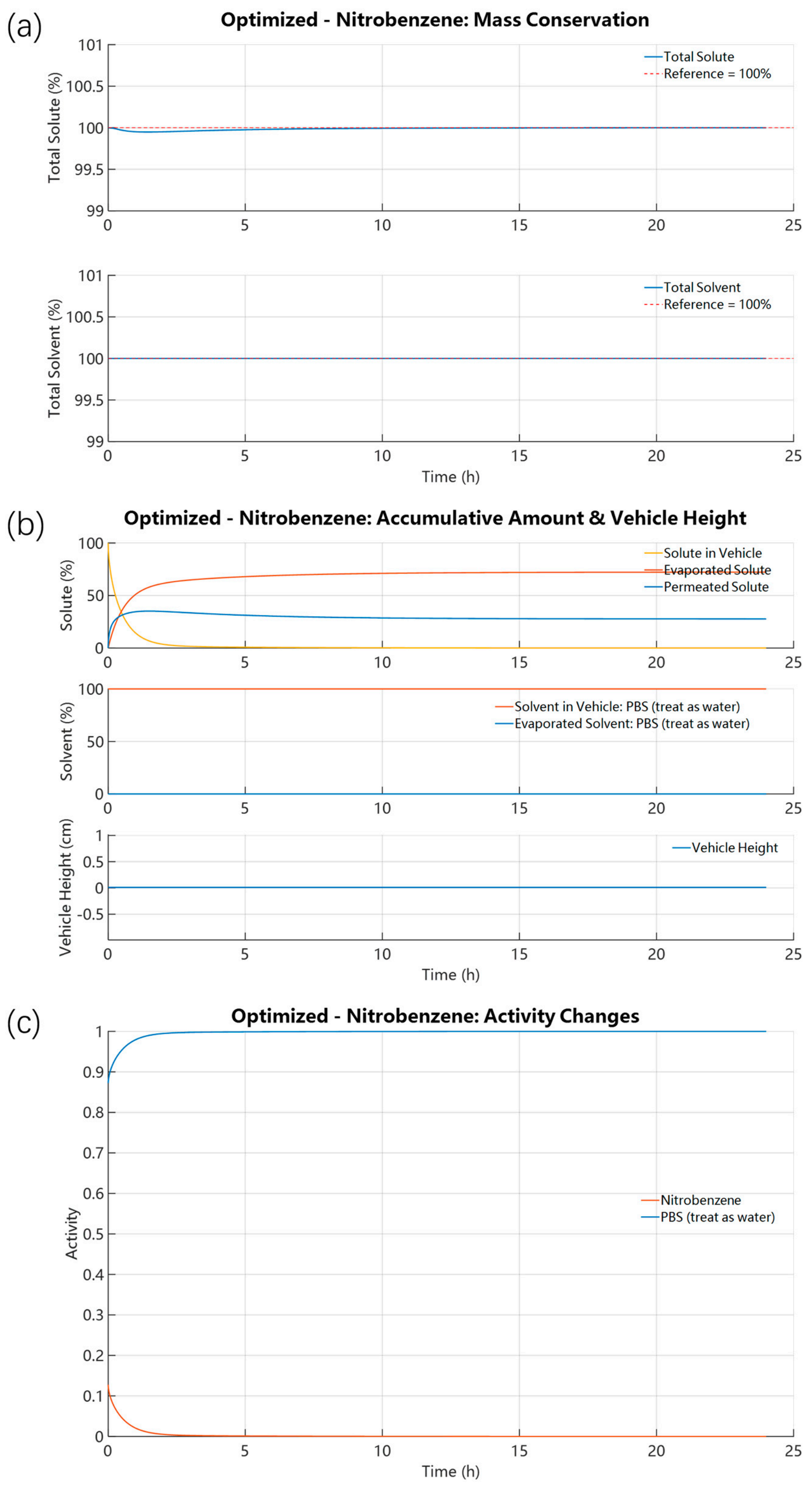

Figure S4 Solvent–solute behaviour and vehicle evolution under preliminary $K_{evap,i}$ optimization of Nitrobenzene ($K_{evap,i} = 8.1174\times10^{-11}$ mol·cm⁻²·s⁻¹, $D_{SC,i} = 5.2677\times10^{-10}$ cm²·s⁻¹, $P_{SCw,i} = 4.8645$). (a) Solute and solvent mass conservation trajectories evaluated under the initial calculated value. (b) Spatial distributions of solute and solvent concentrations together with the vehicle height profile across the evaporation region, vehicle layer, skin domains, and receptor fluid. (c) Temporal evolution of solute and solvent activities within the vehicle under the same initial parameter set.
